# Altitude shapes the environmental drivers of large‐scale variation in abundance of a widespread mammal species

**DOI:** 10.1002/ece3.5851

**Published:** 2019-12-14

**Authors:** Mickaël Jacquier, Clément Calenge, Ludovic Say, Sébastien Devillard, Sandrine Ruette

**Affiliations:** ^1^ Univ Lyon Université Claude Bernard Lyon 1 CNRS Laboratoire de Biométrie et Biologie Evolutive Villeurbanne France; ^2^ Unité‐PAD Office National de la Chasse et de la Faune Sauvage Birieux France; ^3^ Cellule d'Appui Méthodologique Office National de la Chasse et de la Faune Sauvage Saint Benoist France

**Keywords:** climate, food resources, landscape, large‐scale, *Mustelidae*, soil features

## Abstract

**Aim:**

Habitat quality and heterogeneity directly influence the distribution and abundance of organisms at different spatial scales. Determining the main environmental factors driving the variation in species abundance is crucial to understand the underlying ecological processes, and this is especially important for widely distributed species living in contrasting environments. However, the responses to environmental variation are usually described at relatively small spatial scales. Here, we studied the variation in abundance of a widely distributed mustelid, the European badger (*Meles meles*), across France.

**Location:**

The whole metropolitan France.

**Methods:**

We used (a) direct detections of 9,439 dead and living badgers, from 2006 to 2009, to estimate badger relative abundance in 703 small agricultural regions of metropolitan France and (b) a Bayesian modeling approach to identify the main environmental determinants influencing badger abundance.

**Results:**

Despite a continuous distribution of badger in France, we found large variation in badger abundance between regions, explained by environmental factors. Among a set of 13 environmental variables, we demonstrated that badger abundance in lowlands (<400 m a.s.l.) was mostly driven by biotic factors such as potential food resources (earthworm abundance and fruit crops) and forest fragmentation. Conversely, in mountainous areas, abiotic factors (i.e., soil texture and climate) drove the variation in badger relative abundance.

**Main conclusions:**

These results underline the importance of mapping the abundance of wildlife species based on environmental suitability and highlight the complexity of drivers influencing species abundance at such large spatial scales. Altitude shaped the environmental drivers (biotic vs. abiotic) that most influenced relative abundance of a widespread species. In the case of badger, such abundance maps are crucial to identify critical areas for species management as this mustelid is a main wild vector of bovine tuberculosis in several countries.

## Graphical abstract

We used direct detections of 9,439 badgers *Meles meles*, to estimate badger relative abundances in 703 small agricultural regions of metropolitan France and a Bayesian modeling approach to identify the main environmental determinants influencing badger abundances. Despite a continuous distribution of badger in this country, we demonstrated that badger abundances in lowlands were mostly driven by biotic factors such as potential food resources and forest fragmentation, while in mountainous areas, abiotic factors drove the variations in badger relative abundance. These results underline the importance of mapping the abundance of wildlife species based on environmental suitability and highlight the complexity of drivers influencing species abundance at large spatial scale.

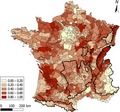

## 1 INTRODUCTION

Distribution and abundance of species are determined by the interaction of ecological processes (such as movement, reproduction, or social interactions) and environmental factors (such as resource availability and habitat configuration; Brown, 1984). Understanding how a species' abundance is likely to vary over space and time helps to plan sound biological management strategies and to identify areas requiring conservation/management attention (Kaiser, 1997). Spatial and temporal environmental heterogeneity can determine the population trends of wild populations, either a population decrease or extinction, or the ability of some species to become overabundant. Species interactions and environmental factors are usually the most important factors limiting distribution and abundance (Boyce et al., 2016; Hoffmann & Blows, 1994), but may act at different temporal and spatial scales, environmental factors being preponderant at large scales (Byrne, Fogarty, O'Keeffe, & Newman, 2015).

Abundance can be highly variable within a species distribution range in response to environmental gradients (Morrison et al., 2006). Areas with high environmental suitability tend to support larger populations (Weber, Stevens, Diniz‐Filho, & Grelle, 2017) because of increased favorability of local conditions (e.g., climatic conditions or available food resources), in particular when ecological requirements are met (Hutchinson, 1957). For example, in an assemblage of freshwater fish, species abundance was associated with species' environmental preferences in the river (e.g., stream size and water clarity; Taylor, Winston, & Matthews, 1993). Landscape characteristics and trophic resource availability can also influence spatial variation in abundance in other taxa such as carnivorous mammals (Pita, Mira, Moreira, Morgado, & Beja, 2009), but to fully understand the habitat driving forces linked to animal abundance, we need better knowledge on how qualitative and quantitative aspects of environmental factors impact the spatial variation in abundance (Morris, 1987).

Identifying patterns of covariation between abundance and environmental factors at the macroscale, that is, over all or at least a large part of the distribution area, requires both a very large amount of occurrence data that allows the estimation of species abundance, and information about environmental predictors distributed over the same large area. However, data of spatial variation in abundance for a species within its distribution range, or at least over a large area far beyond the population scale, are difficult to obtain. Consequently, predictions of spatial abundance patterns over large areas remain scarce for diverse taxa (Sagarin, Gaines, & Gaylord, 2006). Most field and statistical methods rely on complex designs to estimate local absolute abundance (e.g., distance sampling approaches or mark–recapture methods) and are inapplicable or too expensive to be considered for abundance estimation in large spatial scale studies. The relative abundance estimates allow us to study the species over broad spatial scales, under the assumption that the population index is proportional to the population density, and the probability of “detection” for animals in the survey is known (Pollock et al., 2002).

Among mammals, the badger *Meles meles* appears to be a good model species to investigate how environmental factors shape the pattern of variation in abundance over a large spatial scale due to its widespread distribution (Johnson, Jetz, & Macdonald, 2002; Newton‐Cross, White, & Harris, 2007). This nocturnal medium‐sized carnivore, distributed throughout temperate Eurasia, can occupy a large range of biomes, such as woodlands, forests, and arid or mountainous landscapes to a lesser extent (Griffiths & Thomas, 1993). Badger social structure and population abundance are thought to be driven mainly by sett or trophic resource dispersion/availability (Johnson et al., 2002; Kowalczyk, Zalewski, Jedrzejewska, & Jedrzejewski, 2003), which are both driven by landscape patterns (Hammond, McGrath, & Martin, 2001). Characterized by an omnivorous diet, the species can use a wide range of food items including plants (e.g., fruits) and animals such as earthworms or insects (e.g., beetles) depending on the temporal variation of resources availability (Cleary, Corner, O'Keeffe, & Marples, 2011; Kruuk, 1978). The badger is therefore a generalist or opportunist feeder (Roper, 1994). Several environmental determinants have been proposed to explain variation in badger abundance, using predicted sett abundance in large‐scale studies, among which food resource availability, landscape patterns, and climate were the most important drivers (Acevedo et al., 2014; Etherington, Ward, Smith, Pietravalle, & Wilson, 2009; Reid, Etherington, Wilson, Montgomery, & Mcdonald, 2012). A previous study showed that habitat fragmentation due to forest loss affected badger sett density in Spain and led to population isolation (Virgós, 2001). However, using the density of badger setts as an abundance proxy at large scale might be irrelevant due to social group size variation in this species, within seasons (Revilla & Palomares, 2002), or with trophic resources availability (Da Silva, Woodroffe, & Macdonald, 1993). Badger absolute abundance and densities are not known in France, but a recent study has estimated the badger relative abundance (i.e., the actual abundance multiplied by an unknown constant) in each small agricultural region (SAR) of France (Calenge et al., 2015). How these spatial variation in relative abundance over SARs correlate with environmental variation remains unknown.

In the present study, we aim at determining the environmental factors driving spatial variation in badger relative abundance at the scale of all metropolitan France. We first estimated relative badger abundance per SAR over France during 4 years, that is, 2006–2009, using a Bayesian modeling approach expanding previous work of Calenge et al. (2015). Second, we investigated the main ecological drivers of badger relative abundance variation among a set of relevant environmental variables. Although badger is present throughout France, we expected local abundance to be linked to the large variation within the country in terms of agricultural practices, human pressure, and forest cover, and leading to quantitative and qualitative habitat variation between regions. Based on the scientific literature, we predicted that relative badger abundance would be positively correlated with the availability of different potential food resources, such as earthworm abundance (described as the most frequent item in the badger's diet; Kruuk, 1978; Neal & Cheeseman, 1996), which varies widely between spatial units in France (Rutgers et al., 2016). We also expected higher relative badger abundance in woody lowland environments, as this climatic zone offers both the highest level of food resources (Mysłajek et al., 2016) and suitability for sett installation, with favorable mosaic landscapes (Cresswell, Harris, Bunce, & Jefferies, 1989; Kruuk, 1989) including small agricultural fields (i.e., pastures) and forest patches.

## 2 METHODS

### 2.1 Badger data collection

Direct observations of badgers (*M. meles*), that is, presence data, were collected by wildlife protection officers from the French National Hunting and Wildlife Agency (i.e., ONCFS). This organization implements the “small carnivorous logbook program” (SCSL), a program conducted over the whole of metropolitan France, and which asks wildlife protection officers to report all dead and living animals belonging to small carnivorous species (in particular *Mustelidae*) that they randomly meet during their fieldwork (Calenge et al., 2015). Detections of dead or living animals were recorded mainly along roads, when the officers were driving by car (e.g., police patrol missions). Although this program began in 2001 and is still running, we restricted our dataset to 4 years of detections, between 2006 and 2009. Because we needed to make reasonable the assumption of constant relative abundance over the study period, we had to define a shorter study period. Moreover, due to several logistics constraints in recent years (smaller number of officers, smaller budget for gasoline, etc.), the program has collected a much smaller volume of data in recent years. The years 2006–2009 corresponded to the period with the largest number of detections by the data volume.

The spatial unit of interest in our study is the small agricultural region, which is delimited by the intersections between “large agricultural regions” (*n* = 429 in metropolitan France) and administrative departments (*n* = 90). Large agricultural regions correspond to a group of neighboring administrative units (i.e., municipalities) characterized by similar landscapes, agricultural practices, and activities (for further details see http://www.agreste.agriculture.gouv.fr/definitions/zonages). These intersections define 703 homogeneous SARs in metropolitan France (mean = 766.7 km^2^; *SE* = 722.2 km^2^) and for which variation in relative abundance was studied.

### 2.2 Environmental variables

Each SAR was characterized by 13 environmental variables that were grouped into five categories describing landscape, terrain features, potential food resources, climate, and the urbanization level (Table 1). We considered a single value for each variable per SAR for the entire study period. Environmental variables were compiled as shapefile GIS layers using GRASS GIS® software version 7.0.3 (GRASS Development Team, 2016). More information about the choice of these variables, their sources and calculation methods, are given in Appendix S1. Each selected continuous variable was standardized (common mean equals to 0, and common standard deviation equals to 1) to provide comparable effect sizes in the modeling procedure.

**Table 1:** Environmental variables (*n* = 13) calculated for each spatial unit (i.e., small agricultural region) in metropolitan France

Variable name	Description	Unit	Source
Landscape			
*Edge*	Sum of the lengths (m) of all edge segments, divided by total vegetation area (m^2^)	m/m^2^	BD TOPO Vegetation 2015 (IGN^a^)
*Dist*.	Mean distance to the nearest vegetation patch	m	BD TOPO Vegetation 2015 (IGN^a^)
Soil features			
*VRM*	Mean vector ruggedness measure (VRM) of terrain		BD ALTI 2011 (IGN^a^)
*Texture*	Mean index of dominant surface textural class derived from clay, silt, and sand topsoil maps (measured in five categories: from 0 = coarse to 5 = fine)	Index from 0 to 5	BDGSF 1998^b^
*Depth*	Mean index of depth class of an obstacle to roots (measured in 4 categories: 1 = no obstacle to roots between 0 and 80 cm, 2 = obstacle to roots between 60 and 80 cm depth, 3 = obstacle to roots between 40 and 60 cm depth, 4 = obstacle to roots between 0 and 40 cm depth)	Index from 1 to 4	BDGSF 1998^b^
Potential food resources			
*Earthworm*	Predicted median earthworm abundance	Ind./m^2^	Rutgers et al., 2016, c
*Pasture*	Percentage of permanent pastures surface	percentage	RPG 2009^d^
*Maize*	Percentage of maize crop surface	percentage	RPG 2009^d^
*Fruit*	Percentage of orchards and vine crop surface	percentage	RPG 2009^d^
Climate			
*Temperature*	Average monthly temperature of current climate	°C	WorldClim 1.4^e^
*Precipitation*	Average monthly rainfall of current climate	mm	WorldClim 1.4^e^
*Alpi.*; *Conti.*; *Coastal*	From principal component analysis (Appendix S1)		WorldClim 1.4^e^
Anthropic pressure			
*Urban*.	Percentage of urbanized area (classes: 11; 121; 123; 124)	percentage	CORINE Land Cover 2015^f^

a “Institut National de l'Information Géographique et Forestière”, geographical dataset (http://www.ign.fr).

b “Base de Données Géographique des Sols de France”, geographical dataset (www.gissol.fr).

c Digital soil mapping from habitat‐response models (Rutgers et al., 2016).

d "Relevé Parcellaire Graphique", geographical dataset (www.geoportail.gouv.fr).

e Global Climate Data—Free climate data for ecological modeling and GIS (Hijmans et al., 2005).

f CORINE Land Cover 2015, European Environment Agency (http://www.eea.europa.eu).

Landscape variables were mainly related to forest cover representing a favorable environment to setts installation and the presence of badgers. We considered two indices describing the fragmentation of forested areas: (a) the mean distance to the nearest forest patch (from points spaced by 100 m of a dot grid placed throughout France; *Dist*.) and (b) the edge density (*Edge*), as badgers are known to appreciate forest edges (Payne, 2014). We retained three variables describing soil features: (a) terrain ruggedness (vector ruggedness measure: *VRM*; Sappington, Longshore, & Thompson, 2007), (b) dominant surface texture index (between 0 and 5, from coarse to fine texture; *Texture*), and (c) soil depth index (between 0 and 4, from soil with no obstacle to roots to soil with obstacles to roots in the first 40 cm; *Depth*). All three related to the ability to dig and build a sett. Sloped areas are attractive as they aid excavation, and the depth of the soil should allow for sufficient digging (Thornton, 1988). Most relevant potential food resources attractive to badgers were also recorded, that is, the percentage cover of (a) maize (*Maize*) and (b) fruits (i.e., orchard and vineyard; *Fruit*), which are consumed when available (Barea‐Azcón, Ballesteros‐Duperón, Gil‐Sánchez, & Virgós, 2010). (c) The percentage of permanent pasture (any area in which grass or other herbaceous plants have predominated for at least 5 years; *Pasture*) was also taken into account as attractive to badgers, mainly because of the presence of earthworms, which can represent a major part of the species' diet (Do Linh San, 2006; Roper, 2010). We also used (d) the median predicted abundance of earthworms in topsoil (*Earthworm*) from Rutgers et al. (2016), as a proxy of the major potential food resource for badgers (Cleary et al., 2011; Mouches, 1981). To describe the climate of each SAR, we used 24 temperature and precipitation maps of average monthly temperature and rainfall. We applied a principal component analysis using the *ade4* package (Dray & Dufour, 2007) operating in R software (R Development Core Team, 2017) to reduce the number of variables related to climate. The first three principal components were retained measuring, respectively, the intensity of alpine (*Alpi.*), continental (*Conti.*), and oceanic/Mediterranean (*Coastal*) climates of SARs (detailed in Appendix S1). To characterize human pressure, we chose the percentage of urbanized area in each SAR (*Urban.*). Human presence creates disturbance that might negatively affect badger abundance.

A preliminary exploratory analysis indicated that the elevation strongly determined the statistical distribution of other predictors on a national scale. Indeed, mountainous areas are generally correlated with more rocky terrains, a higher forest cover, an alpine climate, steeper slopes, etc. This difference between mountainous and nonmountainous areas will strongly determine any modeling approach to badger density on a national scale. To circumvent this strong leverage effect of the mountainous areas, we divided the 703 SARs into two groups: (a) nonmountainous SARs with an average elevation lower than 400 m (*n* = 531) and (b) mountainous SARs with an average elevation higher than 400 m (*n* = 172). We set the limit of 400 m based on a principal component analysis on all variables, including altitude (further details in Appendix S2). We then modeled the relationship between the predictors and badger abundance in these two groups separately.

### 2.3 Modeling framework

We used a Bayesian variable selection approach to identify the major environmental determinants influencing the relative abundance of badgers. We developed a hierarchical modeling framework extending the modeling approach described in Calenge et al. (2015), to allow both the estimation of the relative abundance of the badger and the identification of variables affecting this relative abundance (for each elevation group of SARs).

The first level of this model was an observation model describing how the relative abundance of the badger was related to the number of badger detections in the SCSL program. This submodel therefore allowed the estimation of the relative abundance of the badger from the number of its detections. This submodel was identical to the approach developed by Calenge et al. (2015) and used the detection of all mustelids species monitored by the SCSL program: (a) the stoat *Mustela erminea*, (b) the weasel *Mustela nivalis*, (c) the polecat *Mustela putorius*, (d) the pine marten *Martes martes*, (e) the stone marten *Martes foina*, and (f) the badger *M. meles*, giving us a better estimation of the sampling effort in the model. We recall briefly the rationale underlying this approach (see Calenge et al., 2015 for further details). Let *N_ijk_* be the total number of animals of the species *i* in each SAR *j* (*j* = 1…*J*) with status *k* (*k* = 1 or 2 for dead and living animals, respectively). *N_ijk_* is assumed to follow a Poisson distribution:

$$N_{\mathrm{ijk}}∼P\left(\lambda_{\mathrm{ijk}}\right)$$

And we supposed as the expectation of this distribution the following model:

$$\lambda_{\mathrm{ijk}}=S_{j}A_{\mathrm{ij}}E_{\mathrm{jk}}P_{\mathrm{ik}}$$

where *S_j_* is the surface area of the *j*th SAR, *A_ij_* is the true density of the species *i* in the SAR *j*, *E_jk_* is the sampling effort in SAR *j* for animals in status *k*, and finally, *P_ik_* is the detection probability of the species *i* in the status *k*. The parameters in the above model are not all identifiable, but Calenge et al. (2015) showed that all the parameters of the following reparametrized model are identifiable:

$$\log\lambda_{\mathrm{ijk}}=s_{j}+a_{\mathrm{ij}}+e_{\mathrm{jk}}+p_{\mathrm{ik}}$$

where *s_j_* is the log surface of the *j*th SAR, *a_ij_* the log‐relative abundance of species *i* in each SAR *j*, *e_jk_* the log‐relative sampling effort for, respectively, dead or alive animals, and *p_ik_* a complex function of the detectability of the species *i* with status *k*. The equivalence between the two models can be demonstrated with the following:

$$s_{j}=\log S_{j}$$

$$a_{\mathrm{ij}}=\log\left(\beta \right)+\log\left(P_{i1}\right)+\log\left(A_{\mathrm{ij}}\right)$$

$$e_{\mathrm{jk}}=\log\left(\frac{E_{\mathrm{jk}}}{\beta }\right)+\log\left(\frac{P_{1k}}{P_{11}}\right)$$

$$p_{\mathrm{ik}}=\log\left(\frac{P_{\mathrm{ik}}}{P_{1k}}\right)+\log\left(\frac{P_{11}}{P_{i1}}\right)$$

This model can be fitted under the assumption that the log‐relative sampling effort is known for dead animals. We assumed that this effort was proportional to the known number of kilometers *Vj* travelled in the *j*th SAR by officers, that is, *e_j_*_1_ = log *Vj* + *β* (see Calenge et al., 2015 for further details on this model and properties of the estimated parameters). Note that this reparametrized model allows in particular the estimation of the desired log‐relative abundance *a_ij_*.

We extended this framework for the particular case of the badger, which is the focus species here. For this species only, we designed a second level in our hierarchical modeling framework: We modeled the relative abundance *a_bj_* of the badger in the SAR *j* as a linear combination of the environmental variables of interest. We used the Bayesian approach for variable selection set by Kuo and Mallick (1998), in order to select the variables that are most likely to explain the relative abundance of badgers among the 13 independent environmental predictors *X_r_* (*r* = 1,…,*R*; *R* = 13). Thus, we supposed that the log‐abundance *a_bj_* of the badger follows a normal distribution of parameters:

$$a_{\mathrm{bj}}∼N\left(\mu_{j},\tau \right)$$

We modeled the expectation of this distribution with the following:

$$\mu_{j}=a_{0}+\sum_{r=1}^{R}\gamma_{r}\beta_{r}X_{\mathrm{jr}}$$

where *a*_0_ is the intercept of the model, and *X_jr_* is the value of the variable *X_r_* (*r* = 1…*R*) for the SAR *j*. The influence of the environmental variable *X_r_* on the mean log‐relative abundance of the badger is supposed to be the result of the combination of two parameters. First, the parameter *γ_r_* is a binary coefficient indicating the presence (*γ_r_* = 1) or absence (*γ_r_* = 0) of the environmental variable *X_r_* in the model. In a Bayesian context, the value of this parameter is supposed to be the realization of a Bernoulli variable with posterior probability *p_r_* that the variable *X_r_* is in the model. Second, the coefficient *β_r_* is the classical regression coefficient associated with variable *X_r_*, can take any real value, and determines the importance of the *r*th variable on the mean log‐relative abundance of the badger when this variable belongs to the model, as in a classical regression model. This approach consists in separating the presence of a variable in a model from its importance and then estimating the probability of the presence of each variable in the model from the data, as suggested by Kuo and Mallick (1998). Note that our model does not account for any residual spatial autocorrelation in the species abundance. Even though accounting for this spatial autocorrelation generally adds to the explanatory power of models, we do not believe that it would be the case here. Indeed, Calenge et al. (2015) included a L2 regularization in their model, accounting for this spatial autocorrelation, and showed that spatial autocorrelation was not very strong in small carnivorous species at this scale and resolution. Besides, the additional level of complexity induced when accounting for spatial autocorrelation may overcomplicate the model and jeopardize the validity of our variable selection approach.

We defined the following uninformative priors on the coefficients of the model:

$$\gamma_{r}∼B\left(0.5\right)$$

$$\beta_{r}∼N\left(0,0.01\right)$$

$$\tau ∼U\left(0.0001,1000\right)$$

Posterior distributions of all the parameters were obtained by Monte Carlo Markov Chain (MCMC) simulations. We ran three chains for an initial period of 1,000 cycles (burn‐in period) and then collected information for the next 300,000 iterations with a thinning of 100. We implemented the MCMC simulations with JAGS software (Plummer, 2018) operating in R software.

From our analyses, we could identify those variables with the largest influence on the relative abundance of the badger and calculate the posterior probability *p_r_* that each variable *X_r_* belongs to the best model (*γ_r_* = 1). We also identify the best models predicting the relative abundance of the badger and calculated the posterior probability *p* (*γ*_1_, *γ*_2_,…*γ_r_*), for each possible combination of the coefficients (*γ*_1_, *γ*_2_,…*γ_r_*). We checked the mixing properties of the MCMC by verifying that the posterior probabilities estimated for the coefficients *γ_r_* were identical across the three chains.

### 2.4 Index of badger relative abundance

For each class of SARs (mountainous and nonmountainous), the procedure of variable selection above allowed us to identify the variables affecting the badger relative abundance *a_bj_* (we retained the predictors with a posterior probability *p_r_* > .5). We therefore fit a final model of *μ_j_* as a linear combination of these variables (*γ_r_* set to 1 for retained variables and 0 for other variables). For each class of SARs, the best models of *μ_j_* allowed the estimation of the coefficient *β_r_* of each selected variable. Badger relative abundance *a_bj_* for each SARs was therefore estimated by *a_bj_* ~ *N* (*μ_j_*, *τ*) and rescaled to represent relative abundance as an index, from 0 to 1.

## 3 RESULTS

### 3.1 Data collection

Wildlife protection officers travelled a total of 82,374,899 km throughout France from 2006 to 2009, leading to the detection of 26,515 animals from six *Mustelidae* species, including 9,439 badgers (35.6% of the detections). Among these detections, 74.2% were roadkill animals and 25.8% were living individuals. We assumed that there were no errors of identification by wildlife officers (a reasonable hypothesis for the badger).

**Figure 1:**
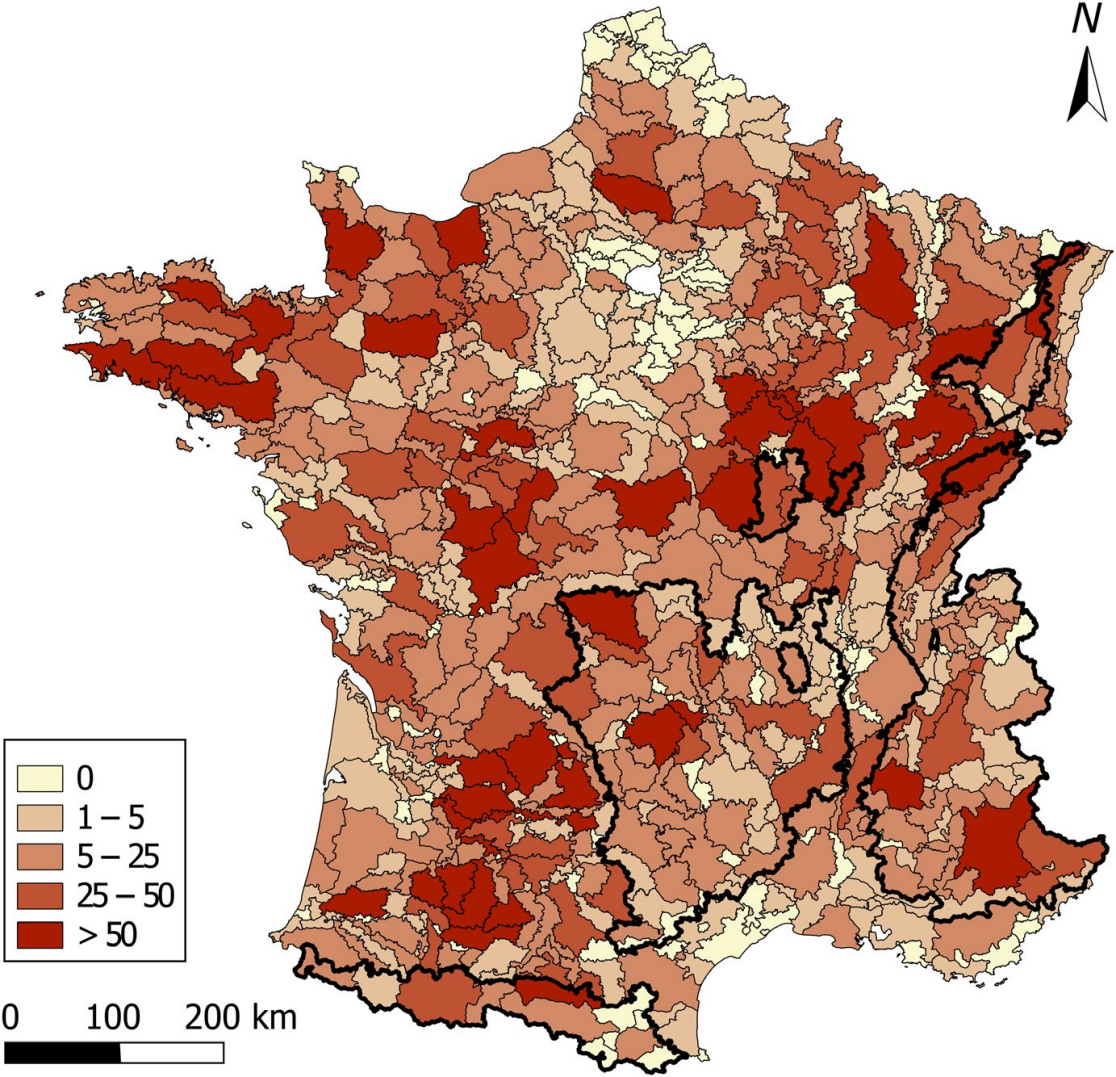
Number of detected badgers (*Meles meles*) in each small agricultural region (SAR) of France from 2006 to 2009 (the inset contains the legend). Bold black lines surround SARs above 400 m mean elevation, representing the limit between mountainous and nonmountainous SARs groups

Among *Mustelidae* species, the badger was the most frequently observed species during the SCSL program, with an average detection of 13.43 animals per SAR (*SE* = 23.36 [min = 0; max = 225]), showing high variability between SARs (Figure 1).

### 3.2 Variable selection models

#### 3.2.1 Nonmountainous SARs group

The relative abundance of badger in nonmountainous SARs (<400 m) was influenced by four environmental variables among the 13, each one being characterized by a posterior probability of being in the true model equal to one (Table 2). The most important variables were related to food availability, such as percentage cover of fruits, pastures, and abundance of earthworms in the topsoil fraction of SARs (Table 2). We found a positive relation between these variables and the relative abundance of badger, showing the importance of resource availability in increasing badger abundance. Edge density had a strong negative influence on the relative abundance of badgers, suggesting that the forest cover should not be too fragmented to support high abundance at this scale.

**Table 2:** Posterior probability *p_r_* of the first eight environmental variables to belong to the best model describing badger relative abundance in metropolitan France, between 2006 and 2009, considering the two small agricultural regions (SARs) groups (i.e., <400 and >400 m elevation)

Variable name	Posterior probability (*p_r_*)	Coefficient estimate (*β_r_*)	95% Credible interval (*β_r_*)
Nonmountainous SARs (<400 m)			
*Earthworm*	1.000	0.461	0.322; 0.600
*Fruit*	1.000	0.400	0.284; 0.518
*Pasture*	1.000	0.541	0.408; 0.680
*Edge*	1.000	−0.407	−0.537; −0.279
*VRM*	.401		
*Dist*.	.060		
*Conti*.	.043		
*Maize*	.039		
Mountainous SARs (>400 m)			
*Texture*	.769	0.355	0.183; 0.523
*Conti*.	.519	0.419	0.247; 0.591
*Maize*	.253		
*Earthworm*	.247		
*Pasture*	.237		
*Urban*.	.195		
*VRM*	.151		
*Depth*	.010		

Note

Associated mean *β_r_* coefficient of selected variables in the best model (i.e., with *p_r_* > .5) is provided along with 95% posterior credible intervals.

Abbreviation: VRM, vector ruggedness measure.

Among the 2^13^ possible combinations of variables, the best model predicting the relative abundance of the badger in low elevations included these four variables. This model was the most often visited by the three MCMC chains, with a posterior model probability *p* (*γ*_1_, *γ*_2_,…*γ_r_*) of .440 (Table 3). The second half of selected models included the effect of other variables characterized by low posterior probabilities, which were not considered here.

**Table 3:** Model structure (i.e., variables combination) and associated posterior model probabilities *p* (*γ*_1_, *γ*_2_,…*γ_r_*) of the first four best models describing badger relative abundance in metropolitan France, between 2006 and 2009, considering the two small agricultural regions (SARs) groups (i.e., <400 and >400 m elevation)

Model structure	Posterior model probability *p* (*γ*_1_, *γ*_2_,…*γ_r_*)
Nonmountainous SARs (<400 m)	
*Earthworm* + *Fruit* + *Pasture* + *Edge*	.440
*Earthworm* + *Fruit* + *Pasture* + *Edge* + *VRM*	.344
*Earthworm* + *Fruit* + *Pasture* + *Edge* + *Dist*.	.044
*Earthworm* + *Fruit* + *Pasture* + *Edge* + *Coastal*	.024
Mountainous SARs (>400 m)	
*Texture* + *Conti*.	.209
*Conti*. + *Earthworm*	.081
*Texture* + *Maize*	.078
*Texture* + *Maize* + *Pasture*	.068

Abbreviation: VRM, vector ruggedness measure.

#### 3.2.2 Mountainous SARs group

Two variables among the 13 relevant environmental variables had the largest influence on relative badger abundance for mountainous SARs (elevation >400 m), with posterior probabilities calculated over all the MCMC iterations *p_r_* > .5 (Table 2). High badger abundance was mostly explained by a higher index of soil texture, which suggested a preference of the badger for fine soil texture. Continental climate also has a positive effect on abundance in mountainous regions. Unsurprisingly, low temperatures and heavy winter precipitation tend to limit badger abundance more in mountainous SARs than in nonmountainous SARs.

The best model predicting the relative abundance of badgers included soil texture and continental climate and was selected by the three MCMC chains, with a posterior model probability *p* (*γ*_1_, *γ*_2_, …*γ_r_*) of .209 (Table 3). Less probable models included mostly the effects of environmental variables related to resource availability (i.e., *Earthworm*, *Maize,* and *Pasture*), with lower posterior model probabilities (*p_r_* < .30); they were not considered here.

### 3.3 Badger relative abundance in France

Bayesian variable selection allowed the identification of four variables affecting the relative abundance of the badger in low elevation SARs and two for high elevation SARs. The final model of *μ_j_* was fitted as a linear combination of these variables (*γ_r_* set to 1) and considering the associated coefficient *β_r_* of each variable on badger relative abundance (estimates and 95% credible intervals in Table 2).

**Figure 2:**
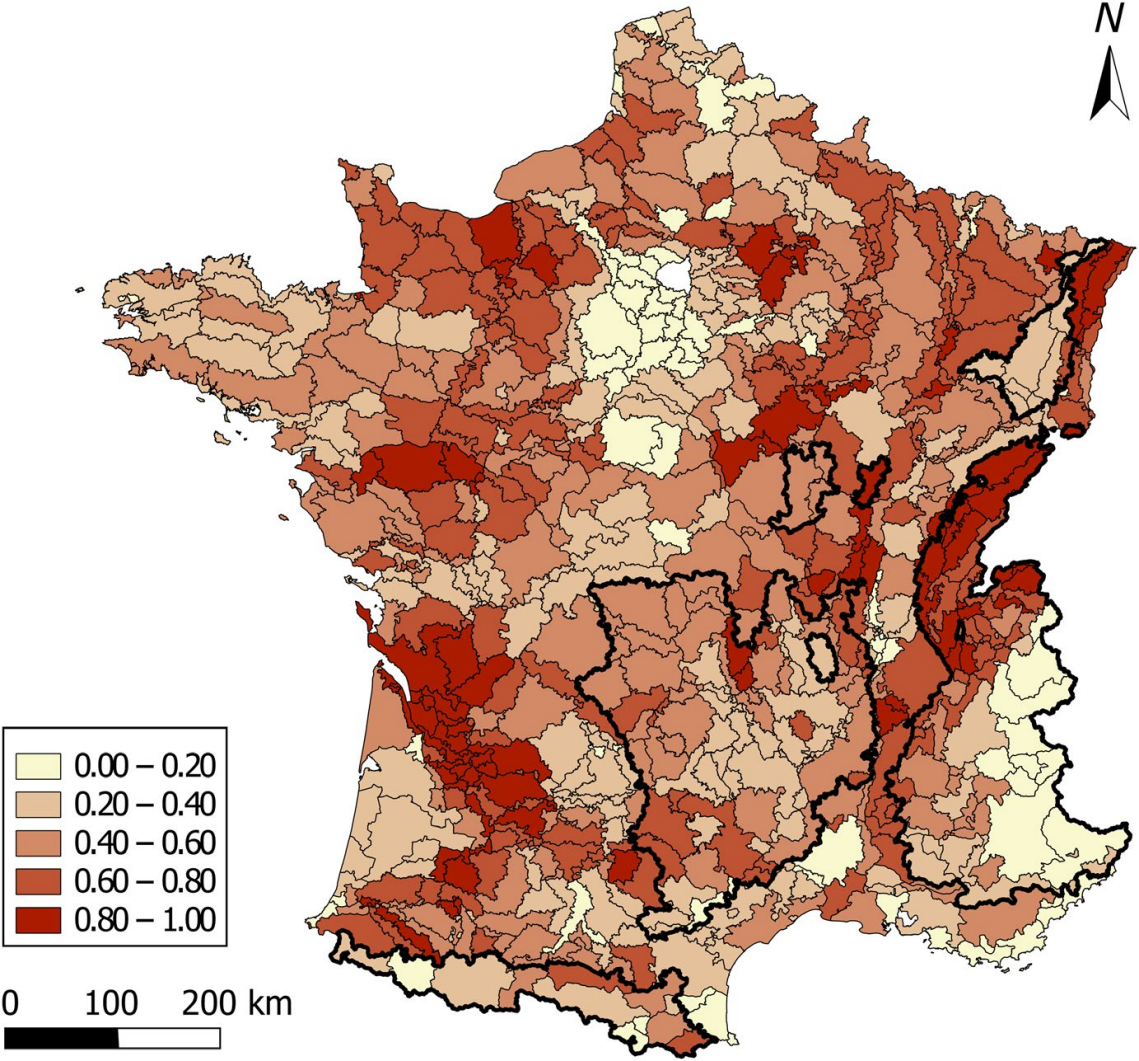
Relative abundance of badgers (*Meles meles*) *a_bj_* as estimated by the best linear combination of selected environmental variables, in the 703 small agricultural region of France for the 2006–2009 period (from 0 to 1: darker areas correspond to higher abundance)

The estimated index of badger relative abundance *a_bj_* (rescaled between 0 and 1) confirmed the presence of the species throughout France, with locally varying abundance between SARs (Figure 2). Badgers were less abundant in the highest mountainous SARs (i.e., the Pyrenees and Alps chains) and in south coastal areas (i.e., Mediterranean rim) which represent areas with low biomass productivity (Revilla, Delibes, Travaini, & Palomares, 1999). Relative abundance estimates *a_bj_* in other parts of low elevation metropolitan France was highly contrasted. Badgers were more abundant in areas of France with the wettest summers and a semicontinental climate (e.g., north‐western and south‐eastern parts of France), and in Brittany where the amount of available food resources is large (e.g., *Earthworm*) despite a high edge density. However, the badger appeared less abundant in the north‐central area of France, where several neighboring SARs had very low relative abundance (*a_bj_* < 0.3). No significant differences in relative abundance were observed between SARs groups (<400 m: mean = 0.50, *SE* = 0.31; >400 m: mean = 0.48, *SE* = 0.27).

## 4 DISCUSSION

In this study, we used direct detections of dead and living animals to investigate the environmental drivers of badger abundance over a large area, that is, metropolitan France, between 2006 and 2009. Despite a continuous distribution of badgers over the country, there is a large variation in badger abundance between SARs, explained by environmental determinants. Our results demonstrated that badger relative abundance was in the same range in both nonmountainous (<400 m) and mountainous (>400 m) groups of SARs, but was driven by different environmental variables. Biotic factors (i.e., food resources) drove badger relative abundance at low elevations, while abiotic factors (i.e., soil texture and climate) played this role at high elevations.

### 4.1 Determinants of variation in badger relative abundance

#### 4.1.1 Nonmountainous SARs group (<400 m)

Among the relevant environmental variables used in our model, those related to potential food resources seemed to be the most important variables supporting higher badger abundance in nonmountainous SARs. Badgers have a broad omnivorous diet, although earthworms are often described as the preferred food resource (Kruuk & Parish, 1982). The results confirmed that landscapes at low elevations with a high abundance of earthworms tended to host higher badger abundance, as well as SARs showing a high proportion of permanent pastures, which are often described as foraging areas for badgers (Hammond et al., 2001; Reid et al., 2012). Permanent pastures are moreover often presented as areas with a high biomass of earthworms (Da Silva et al., 1993). The presence of badgers in lowland areas of France is also favored by arable lands supporting high trophic resources such as orchards and vineyards, which is consistent with previous studies on badgers (Barea‐Azcón et al., 2010; Rosalino, Macdonald, & Santos‐Reis, 2005). By contrast, the presence of maize fields did not increase (or decrease) the abundance of badgers at the large spatial scale of France, although badgers can use this food resource in their diet (Lanszki & Heltai, 2011; Moore et al., 1999), especially during late summer when food resources are more scarce.

High badger abundance was also associated with less fragmented SARs. Highly fragmented landscapes (i.e., characterized by high edge density values) reduce the availability of suitable sett sites for badgers through loss of forest cover and increase isolation (Virgós, 2001). Badgers may be vulnerable to habitat loss and population fragmentation, mainly because of low dispersal rate (Woodroffe, Macdonald, & Da Silva, 1995). In the northern central part of France, high edge density seems to induce low relative abundance of badgers in these SARs (*a_bj_* < 0.3). However, in Brittany, despite high edge density values, SARs support high badger relative abundance (*a_bj_* > 0.6). The presence of arable lands and a high percentage of pastures with high‐predicted earthworm abundance seem to compensate for habitat fragmentation. Results suggest that the highest abundance of badgers in lowlands occurs in favorable connected landscapes, with a variety of trophic resources, suitable for this opportunistic feeder.

#### 4.1.2 Mountainous SARs group (>400 m)

The fine soil texture and continental climate (characterized by moderate precipitation trends, concentrated mostly in the warmer months) appeared as the main environmental drivers to increase badger abundance in this SARs group. These two variables seem linked to a gradient of elevation, from favorable lowlands composed of finer soil texture (allowing digging for sett installation; Obidziński, Pabjanek, & Mędrzycki, 2013) and continental climate, to mountainous areas composed of rocky soils and alpine climate (characterized by high precipitation trends and lowest annual temperatures). According to this gradient, SARs above 1,000 m of mean altitude (*n* = 45) are indeed characterized by very low relative abundance of badgers (mean = 0.31, *SE* = 0.24), due to an unsuitable environment (i.e., long duration of snow cover and low winter temperatures). As explained before, the SAR elevation variable was not included in our Bayesian model due to the high correlation between environmental variables and elevation (e.g., terrain ruggedness and edge density). Although SARs have been separated into two distinct groups (i.e., <400 and >400 m), the effect of elevation still seems important here and confirmed that elevation was the main driver of badger abundance for highest elevation SARs.

While biotic factors (such as earthworm biomass or the presence of fruit crops) drove badger abundance in lowland areas, lower variation in—and lower levels of—food supply in mountainous areas may explain why these biotic factors do not influence badger abundance variation at high elevations. Conversely, climate and soil structure played a preponderant role in shaping badger abundance above 400 m altitude, probably by imposing constraints on sett locations.

### 4.2 SARs scale effect and model accuracy

Because the data were collected mostly along roads, the spatial distribution of detections is not independent of roads locations. However, we do not think that roads locations resulted in a strong bias here. Indeed, on a large scale, the roads are very dense and well distributed throughout France. Moreover, the role of wildlife protection officers is to enforce the law uniformly over their territory (whatever the density of roads), resulting in a sampling effort mostly dependent on the distance travelled by the officers (i.e., our measure of effort) and not on the spatial distribution of the roads. On a small scale, it is important to note that our sampling units (i.e., SARs) are characterized by a homogeneous landscape, tending to limit such bias within a unit. Even though the density of a species is probably different along the road and in the surrounding habitat, the detectability parameters in our model take this difference into account provided that it is constant over SARs (e.g., density along the roads always 50% smaller than in the surrounding habitat).

We assumed that environmental variables, averaged over each SAR, were distributed uniformly over these spatial units in our model. However, SARs' areas ranged from 11.2 to 4,404.3 km^2^ (mean = 766.7 km^2^; *SE* = 722.2 km^2^) and might present within‐SAR environmental variability, especially inside the mountainous SARs group (i.e., >400 m) where the within‐SAR variation in elevation is high (mean elevation variation = 1,293.6 m, *SE* = 771.4 m). In mountainous SARs group for example, this might induce differences in abundance within SARs, with higher abundance of badgers in the foothills, as these climatic and environmental areas offer the richest trophic resources, and the highest availability of suitable sett sites due to a finer soil texture (Mysłajek et al., 2016). Although such local variation in abundance likely occurs within SARs, it should not drastically change the relative position of the SARs along the observed *a_bj_* continuum: The within‐SAR abundance variation should be smaller than the between‐SARs abundance variation.

With a low intrinsic population growth rate (Anderson & Trewhella, 1985), badger abundance was expected to be constant during our study period (i.e., between 2006 and 2009). Environmental variables were also expected to be constant during the 4‐year period at the scale we considered in our study. Indeed, given that SARs are large spatial units (767 km^2^ on average), the forest and crop surfaces will likely remain constant over 4 years, even though it changes at much local scale. Similarly, soil features and global climatic variables remain constant within SARs on such a short time scale. However, some environmental variables may vary seasonally (e.g., earthworm, Cleary et al., 2011). Resource availability or climate could vary across seasons and may not have a constant effect on badger abundance. For example, considering trophic resources, the presence of maize fields in a SAR induced a temporary availability of a potential food resource, corn being available for badgers only during late summer. These seasonal variations are constant across years in a given SAR and will not affect estimated relative abundance over a 4‐year period. Pursuing this study over a much longer term could allow us to test how between years variation in food productivity and/or climatic conditions dynamically influences badger abundance. Unfortunately, to date, most of the environmental data are not available on a yearly basis.

## 5 CONCLUSION

Different environmental factors drove the badger abundance depending on the elevation. While biotic factors acted in lowland parts of France, abiotic factors were associated with spatial variation in relative abundance in higher elevation areas. This calls for the use of spatial replications in habitat–resource–occurrence or abundance studies over heterogeneous landscapes. Mapping the relative abundance of wildlife species based on environment suitability can be used to develop conservation strategies for endangered populations (Gibson, Wilson, Cahill, & Hill, 2004) or to evaluate risks of a species to become overabundant, especially for species acting as wild reservoir hosts for infectious disease (Bessell, Orton, White, Hutchings, & Kao, 2012). The presence of high abundance of badgers in some parts of France (e.g., mean *a_bj_* = 0.72 in Dordogne) can, for example, help to identify areas of potential risk for zoonotic transmissions, such as bovine tuberculosis, which has recently been found in cattle and badgers in France (Payne et al., 2013).

Beyond environmental drivers, interspecific competition can be a major process shaping wildlife communities (Schoener, 1983). However, interactions within mammal communities have only rarely been studied at large scale, and such an approach using relative abundance may allow us to study the coexistence of species. Interactions between mesocarnivores can influence competitive relationships (Tammeleht & Kuuspu, 2018) or abundance, as shown by the culling of European badgers for disease control in England, which was associated with increases in red fox *Vulpes vulpes* densities (Trewby et al., 2007). Detections of all mustelid species monitored by the SCSL program could thus allow studying the relationships between the relative abundance of these species in France and interactions within the mustelid community.

## CONFLICT OF INTEREST

The authors declare that they have no conflict of interest.

## AUTHOR CONTRIBUTIONS

SD and SR designed the study. MJ and CC conducted the statistical analyses. MJ drafted the manuscript. MJ, CC, LS, SR, and SD critically revised the manuscript. All authors approved final manuscript for submission.

## PERMITS

The fieldwork was made by 1,500 qualified officers working for the ONCFS, commissioned by the French Minister of Ecology, which allowed them to manipulate dead animals, according to French legislation. Data collection for the SCSL program was a part of their job, so their written or verbal consent for this study was not required. The director of the ONCFS approved the SCSL program.

## Data Availability

The data that support the findings of this study (i.e., animal detections and kilometers travelled with cars) are available in a public accessible data repository: Dryad: https://doi.org/10.5061/dryad.8pk0p2nhz.
