# Supplementary material for: Altitude shapes the environmental drivers of large‐scale variation in abundance of a widespread mammal species
Source: Ecol Evol. 2019 Dec 14;10(1):119–30. doi: 10.1002/ece3.5851 (PMC6972803; doi:10.1002/ece3.5851)
Supplement: Supplementary file 1 [file ECE3-10-119-s001.docx]

**SUPPORTING INFORMATION**

**APPENDIX S1**

This supplementary material provides more information about the calculation of environmental variables used in the Bayesian variable selection model. The 703 spatial units (i.e. small agricultural regions: SARs) were characterised using 13 variables grouped in five categories describing: landscape, terrain features, potential food resources, climate and finally urbanisation level (Table 1 in the main paper).

*Variables related to landscape (n = 2)*

Two variables were related to the landscape in the SARs. We first defined the edge density to characterise each spatial unit for the opportunity of refuge for badgers (*Edge*; Fig. S1.1), as the sum of the lengths of all edge segments (m) in SARs, divided by total vegetation area (m²). In addition, we estimated the mean distance in meters to the nearest vegetation patch from each point of a single grid spaced by 100 m placed throughout France (*Dist.*; Fig. S1.1), traducing isolation of vegetation patches. These two variables were calculated from the GIS database BD TOPO Vegetation 2015 from the French National Geographic Institute (IGN; [www.ign.fr](http://www.ign.fr)).

*Variables related to terrain features (n = 3)*

We considered three variables characterising terrain features in the SARs. We first defined terrain ruggedness as the mean vector ruggedness measure (*VRM*; Fig. S1.2), which measures local variation in terrain more independently of slope than other metrics like the terrain ruggedness index TRI (Sappington, Longshore, & Thompson, 2007). This variable was calculated from the GIS database BD ALTI 25 m (IGN). We also derived a mean index of dominant surface textural class using clay, silt and sand topsoil maps (measured in 5 categories: from 0 = coarse to 5 = fine; *Texture*; Fig. S1.2), and another mean index of soil depth class (measured in 4 categories: 1 = no obstacle to roots between 0 and 80 cm, 2 = obstacle to roots between 60 and 80 cm depth, 3 = obstacle to roots between 40 and 60 cm depth, and/or 4 = obstacle to roots between 0 and 40 cm depth; *Depth*; Fig. S1.2). These last two variables were calculated from the French Geographical Soil Database BDGSF 1998 ([www.gissol.fr](http://www.gissol.fr)).

*Variables related to potential food resources (n = 4)*

We considered four variables characterising the presence of potential food resources for badgers in SARs, by computing the percentage of maize crops surface area (*Maize*; Fig. S1.3), orchards and vine crops surface area (*Fruit*; Fig. S1.3) and permanent pastures surface area (defined as area in which grass or other herbaceous plants has been predominant for five years; *Pasture*; Fig. S1.3) for each spatial unit. Pastures are attractive to badgers due to the high biomass of earthworms, often described as the principal component of their diet (Roper, 2010). These three variables were computed based on the land registration system (RPG 2009; www.geoportail.gouv.fr) geographical dataset.

A fourth variable of predicted median earthworm abundance (ind. / m²; *Earthworm*; Fig. S1.3) was obtained from the study of Rutgers et al. (2016), which developed a European digital soil mapping of earthworm diversities and abundance from habitat-response models.

*Variables related to climate (n = 3)*

In order to characterise the climate of each spatial unit, we used a set of global climate layers (gridded data) from the free database WorldClim 1.4 (Hijmans et al., 2005). In total, 24 GIS layers with a spatial resolution of about 1 km² were used, describing monthly the mean current precipitation (rainfall in mm) and the mean current temperatures (°C) in France. We reduced the number of variables to 3 composites variables (*Alpi.*, *Coastal* and *Conti.*) using a Principal Component Analysis with the *ade4* package (Dray and Dufour, 2007) operating in R software (R Development Core Team, 2017). The sum of the eigenvalues for these first three axes of the PCA accounted for more than 92% of the total variability.

The first principal axis of this PCA opposed mainly the Alps (characterised by low mean temperatures and abundant rainfalls) and other areas, especially the Mediterranean rim, with high mean temperatures and lower rainfalls (*Alpi.*; Fig. S1.4). The second principal axis opposed, among the non-mountainous areas, the rainiest areas (e.g. Brittany and south-western France) and drier areas (e.g. Central and north-eastern France) in winter (*Coastal*; Fig. S1.4). Finally, the third ACP axis opposed mainly areas with a continental climate (with rainy and hot areas in summer), and areas with milder and drier summers (e.g. north-western France; *Conti.*; Fig. S1.4).

In order to use this climatic information in our Bayesian modelling approach, we calculated a mean value per SARs for each of these three composite variables.

*Variable related to urbanisation level (n = 1)*

A fifth and last group of variables concerned the urbanisation level in SARs. We computed the percentage of urbanised area for each spatial unit using the European landscape GIS database CORINE Land Cover 2015 (www.eea.europa.eu), by grouping classes 111; 112; 121; 123 and 124 (i.e., Continuous urban fabric; Discontinuous urban fabric; Industrial or commercial units and public facilities; Port areas; and Airports respectively). This variable expressed the human pressure in each spatial unit of the study (*Urban.*; Fig S1.5).

**Fig. S1.1**. Cartography of the landscape variables, with: (A) the edge density (in m / m²; *Edge*) and (B) the mean distance to the nearest vegetation patch (in m; *Dist.*), calculated for each 703 small agricultural region in metropolitan France. Colour shades correspond to quartiles of each variable.

(A) (B)


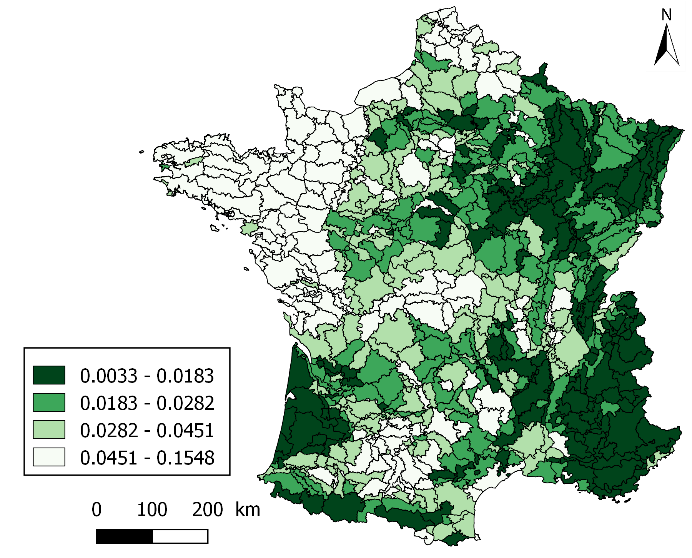

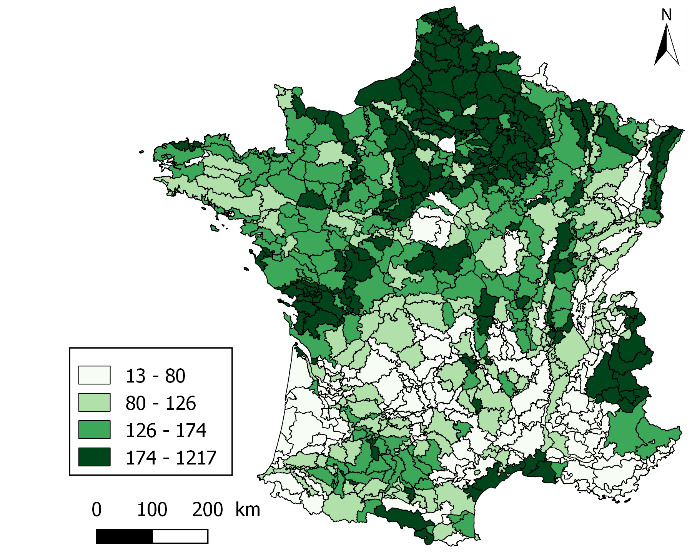


**Fig. S1.2**. Cartography of the soil features variables, with: (A) the mean Vector Ruggedness Measure (in degrees; *VRM*); (B) the mean index of dominant surface textural class (from 0 = coarse to 5 = fine; *Texture*) and (C) the mean index of soil depth class (from 1 = no obstacle to roots to 4 = obstacle between 0 and 40 cm depth; *Depth*), calculated for each 703 small agricultural region in metropolitan France. Colour shades correspond to quartiles of each variable.

(A) (B)


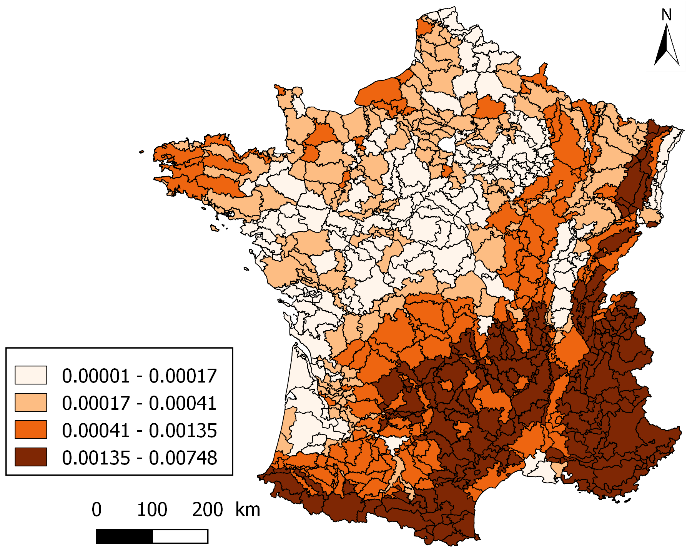

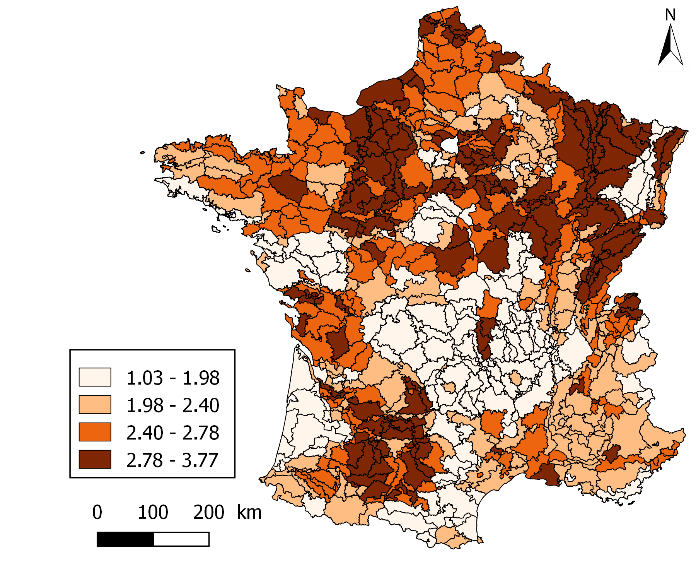


(C)


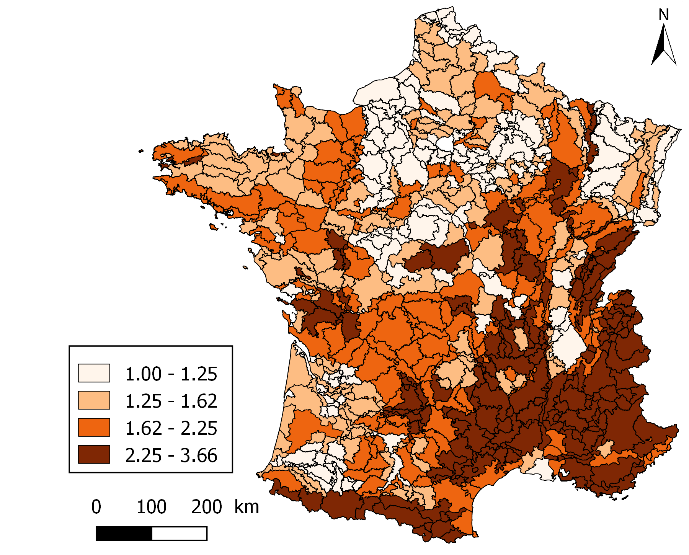


**Fig. S1.3**. Cartography of the potential food resources variables, with: (A) the predicted median earthworm abundance (in ind. / m²; *Earthworm*), (B) the percentage of permanent pastures surface (in %; *Pasture*), (C) maize crop surface (in %; *Maize*) and (D) orchards and vine crop surface (in %; *Fruit*), calculated for each 703 small agricultural region in metropolitan France. Colour shades correspond to quartiles of each variable.

(A) (B)


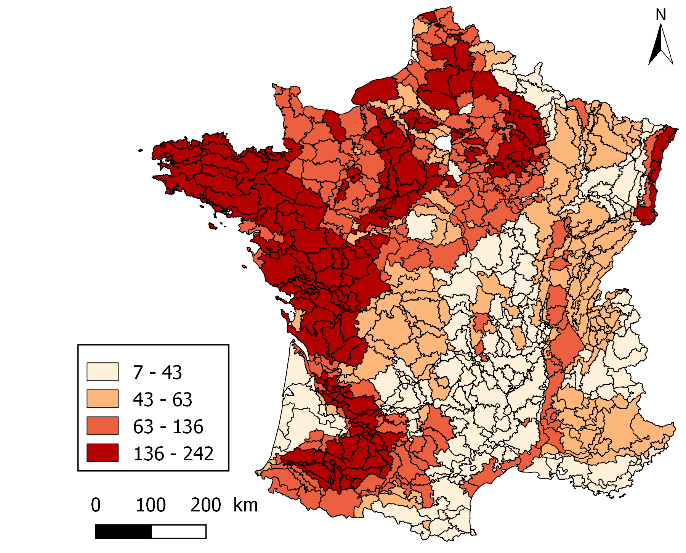

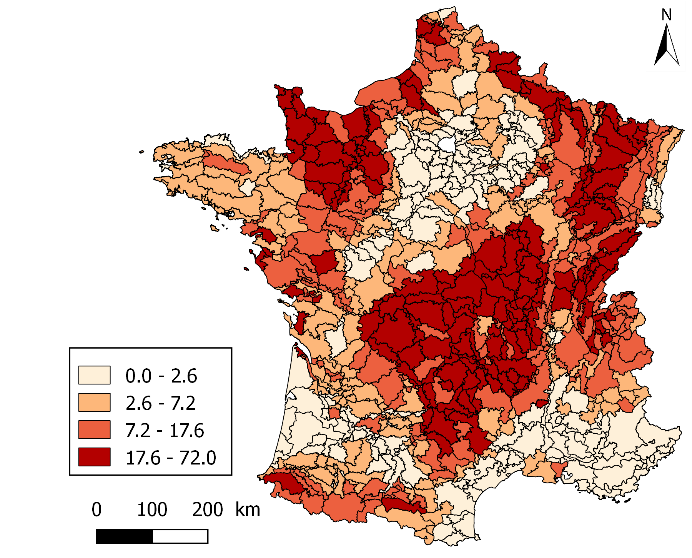


(C) (D)


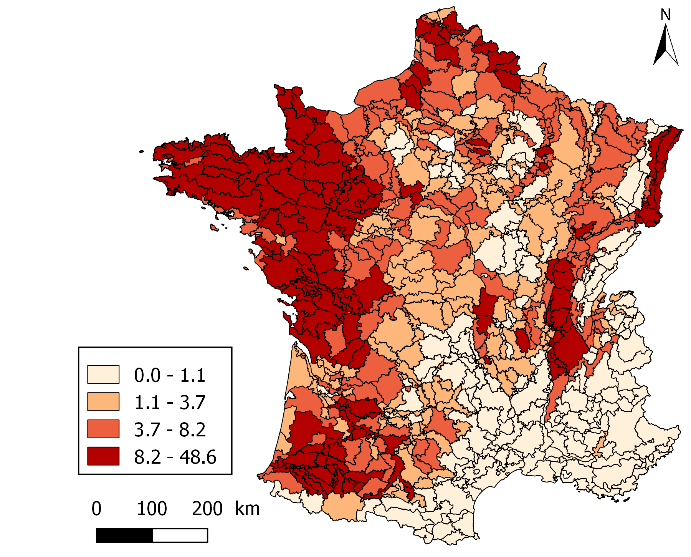

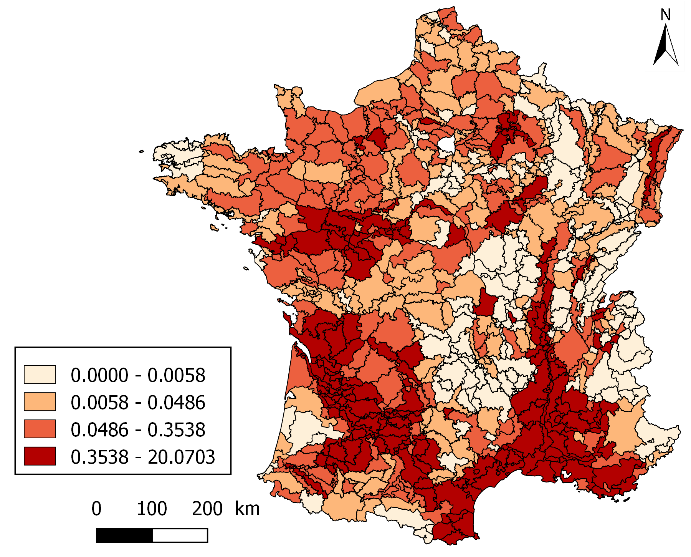


**Fig. S1.4**. Cartography of the climatic variables, with: the (A) first, (B) second and (C) third axis of the Principal Component Analysis (*Alpi.*, *Coastal* and *Conti.* respectively) using the 24 precipitation and temperature GIS layers from WorldClim 1.4.

(A) (B)


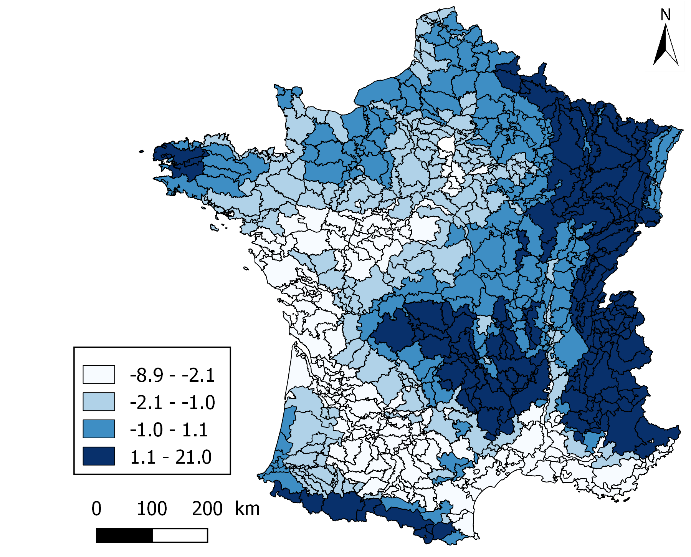

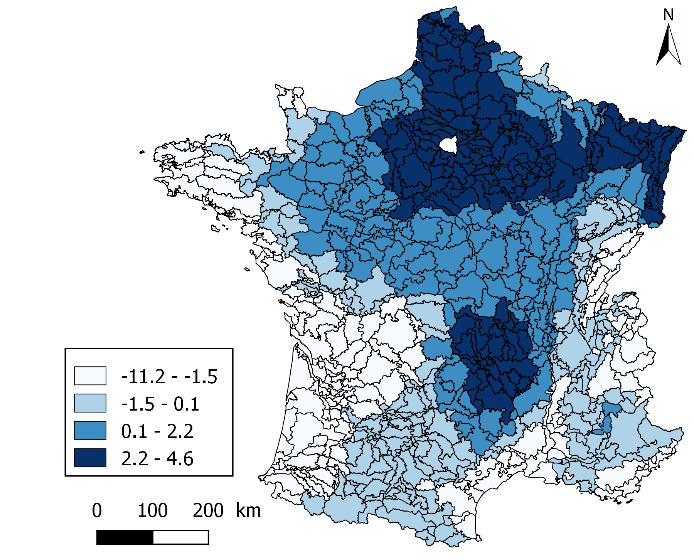


(C)


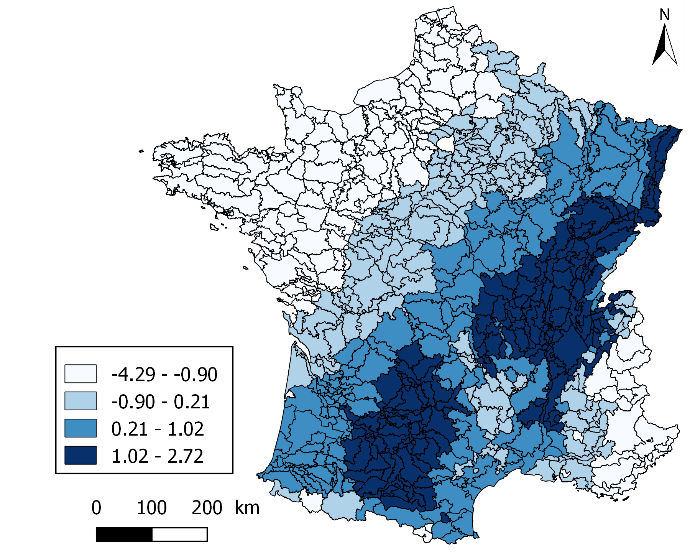


**Fig. S1.5**. Cartography of the anthropic pressure, with the percentage of urbanised area (in %; *Urban.*) calculated for each 703 small agricultural region in metropolitan France. Colour shades correspond to quartiles of the variable.


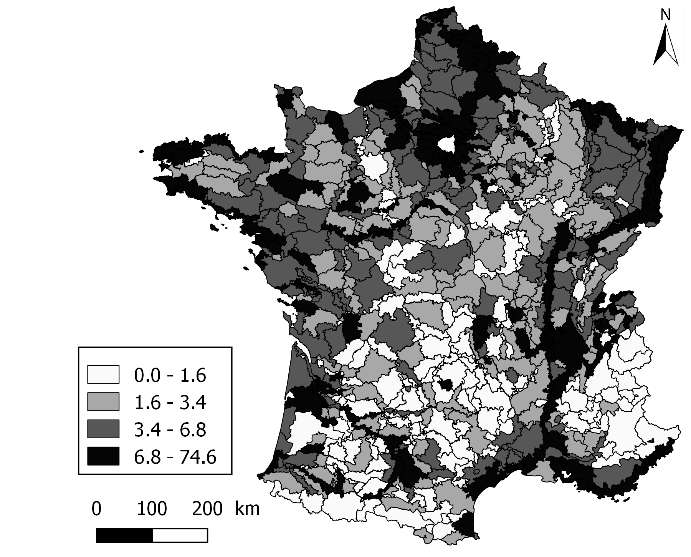


**REFERENCES**

Dray, S., & Dufour, A. (2007). The ade4 package: implementing the duality diagram for ecologists. *Journal of Statistical Software*, **22**, 1–20.

Hijmans, R. J., Cameron, S., Parra, J., Jones, P. G., Jarvis, A., & Richardson, K. (2005). *WorldClim, version 1.3*. University of California, Berkeley.

R Development Core Team 2017. R: A language and environment for statistical computing. R Foundation for Statistical Computing, Vienna, Austria. http://www.R-project.org/

Roper, T. (2010). *Badger*. The New Naturalist Library, Collins, London.

Rutgers, M., Orgiazzi, A., Gardi, C., Römbke, J., Jänsch, S., Keith, A. M., … De Zwart, D. (2016). Mapping earthworm communities in Europe. *Applied Soil Ecology*, **97**, 98–111. https://doi.org/10.1016/j.apsoil.2015.08.015

Sappington, J. M., Longshore, K. M., & Thompson, D. B. (2007). Quantifying landscape ruggedness for animal habitat analysis: a case study using bighorn sheep in the Mojave Desert. *Journal of Wildlife Management*, **71**, 1419–1426. https://doi.org/10.2193/2005-723

**APPENDIX S2**

This document provides more details about the separation of the 703 small agricultural regions (i.e. SARs) into two distinct groups, based on mean SAR altitude.

We carried out a Principal Component Analysis of the table giving the value of the 13 relevant environmental variables in each SAR, using the *ade4* package (Dray and Dufour, 2007) operating in R software (R Development Core Team, 2017). The first axis of this PCA, accounting for more than 30% of the total variability, expressed a strong elevation gradient in France, opposing lowlands on the negative values of this axis, to mountainous SARs with high ruggedness values, and low depth soils (Fig. S2.1). Note that many of our variables of interest were highly correlated with this first axis (earthworm, VRM, depth, etc.). Thus, as a whole, the main structure of the environmental variables identified by this PCA is the elevation gradient (see Fig. S2.2). Modelling badger abundance at the national scale without accounting for this gradient would result in an erroneous interpretation: the variables the most correlated with the first axis would mainly express the elevation gradient, and not necessarily the biotic or abiotic factors of interest.

To avoid this problem, within the 703 SARs, spatial units were separated into two elevation groups. Indeed, we examined the relationship between SAR mean altitudes and the scores of the SAR on the first component of the PCA, and we visually identified 400 m as the best separation between two groups of SARs (Fig. S2.2). We retained as different groups: - SARs with an average altitude lower than 400 m (n = 531); and - SARs with an average altitude higher than 400 m (n = 172). Figure S2.3 illustrates the localisation of non-mountainous and mountainous SARs in France.

**Fig. S2.1**. (A) Eigenvalues of the Principal Component Analysis included the 13 relevant environmental variables selected and (B) the correlation circle of the PCA (2 axis retained).

(A) (B)


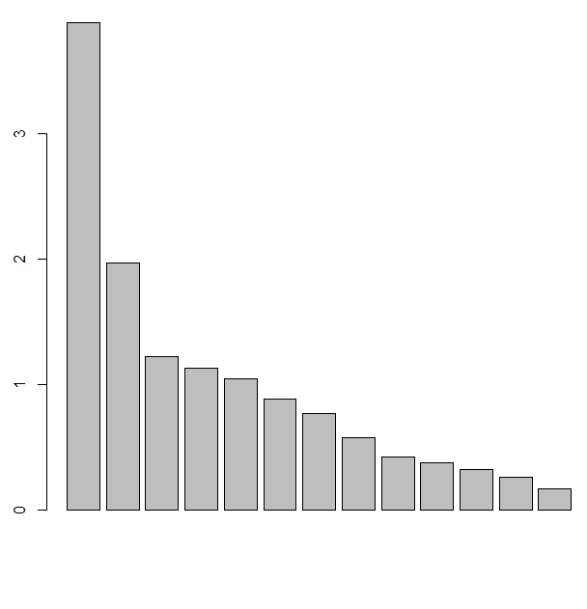

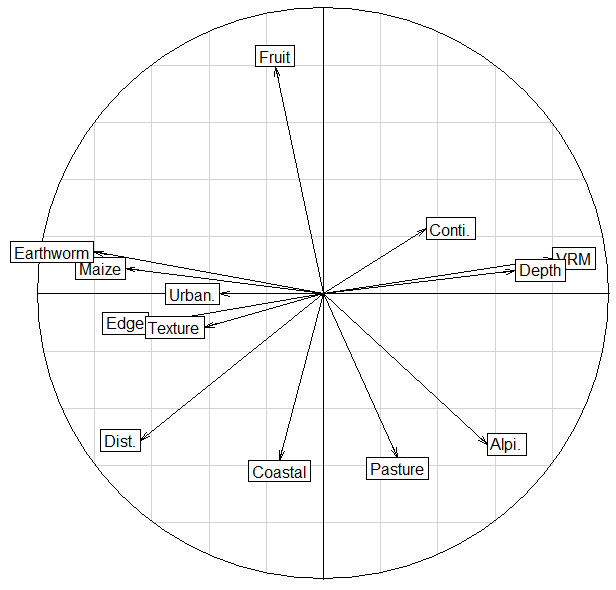


**Fig. S2.2**. Correlation between the SARs’ mean altitudes and the first component (i.e. Dim. 1) of the Principal Component Analysis included the 13 environmental variables. Dotted line indicates the 400 m mean elevation limit.


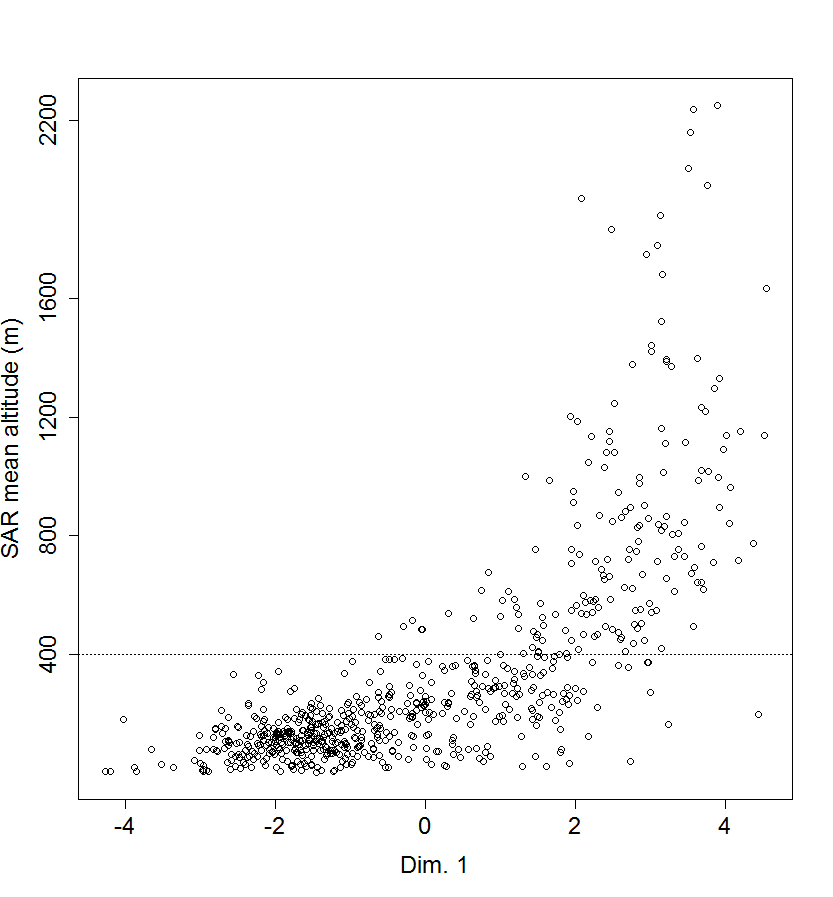


**Fig. S2.3**. Cartography of the 703 SARs in metropolitan France. Black area represents SARs above 400 m mean altitude (n = 172).

**
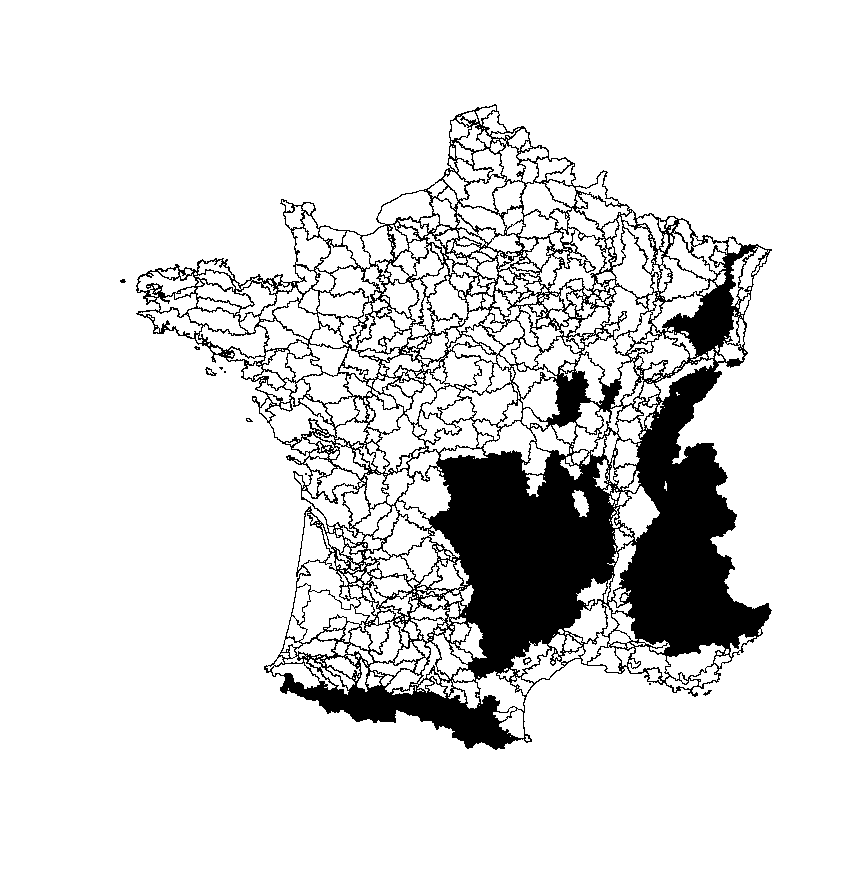
**

**REFERENCES**

Dray, S., & Dufour, A. (2007). The ade4 package: implementing the duality diagram for ecologists. *Journal of Statistical Software*, **22**, 1–20.

R Development Core Team 2017. R: A language and environment for statistical computing. R Foundation for Statistical Computing, Vienna, Austria. http://www.R-project.org/
